# The critical role of PPARα in the binary switch between life and death induced by endoplasmic reticulum stress

**DOI:** 10.1038/s41419-020-02811-4

**Published:** 2020-08-11

**Authors:** Ling Xu, Xiangying Zhang, Yuan Tian, Zihao Fan, Weihua Li, Mei Liu, Jianhua Hu, Zhongping Duan, Ronghua Jin, Feng Ren

**Affiliations:** Beijing Youan Hospital, Capital Medical University, No. 8, XitouTiao Road, Youwai Street, Fengtai District Beijing, 100069 China

**Keywords:** Apoptosis, Apoptosis, Apoptosis, Stress signalling, Stress signalling

## Abstract

Endoplasmic reticulum stress (ER stress) just like a double-edged sword depending on different conditions in the development of multiple hepatic diseases. But the molecular mechanisms of functional conversion during ER stress have not been fully elucidated. In this study, we aim to illustrate the role of PPARα and the subtle mechanism in the functional conversion of ER stress. Tunicamycin (TM) and thapsigargin (TG), as ER stress inducers, were used to induce ER stress in AML12 cells. During the ER stress, qRT-PCR and immunoblotting was used to measure the expression levels of GRP78 and CHOP which show a gradually increasing trend, while PPARα and autophagy was significantly activated in the early stage but was inhibited in the late stage. Moreover, PPARα inhibition by siRNA promoted cell injury in the mild-ER stress and PPARα activation by WY-14643 reduced cell apoptosis in the serious ER stress. In the mild-ER stress with PPARα knocked down, activation of autophagy by rapamycin significantly improved cell survival, in the serious ER stress with PPARα activation, inhibition of autophagy by 3-MA aggravate cell injury. In addition, in the mild-ER stress with PPARα knocked down, CHOP knocked down by siRNA reduced cell apoptosis, in the serious ER stress activated PPARα, CHOP over-expression mediated by lentiviral vector contributed to serious cell injury. Furthermore, C57BL/6 mice was used to induce ER stress with TM intraperitoneal injection, PPARα and autophagy was upregulated in the mild-ER stress while downregulated in the serious ER stress, measured by qRT-PCR and immunoblotting, further confirmed the finding in vitro. Our results firstly demonstrated that PPARα is a key molecule in the functional conversion of ER stress: protective effects in the mild ER stress was mediated by PPARα-autophagy pathway and destructive effects in the serious ER stress was mediated by PPARα-CHOP pathway.

## Introduction

In eukaryotic cells, the endoplasmic reticulum (ER) is responsible for multiple functions, such as protein folding and modification, lipid synthesis, and calcium storage and release. Conditions that may disturb ER homeostasis include surges in protein synthesis, energy deprivation, and imbalances of ER calcium levels. These conditions lead to accumulation of unfolded or misfolded protein in the ER lumen, which is known as endoplasmic reticulum stress (ER stress). Hepatocytes are rich in ER content because of their high protein synthesizing capacity; therefore, ER stress-induced hepatocyte injury plays an important role in the pathogenesis of various liver diseases. Some studies have found that ER stress is involved in the progression of nonalcoholic fatty liver disease^1^. Further, rapid replication of hepatitis C virus and accumulation of viral protein in the ER has been shown to trigger ER stress to promote hepatocytes damage^2^. Finally, arylating quinones have been used as clinical analgesics to induced liver injury, and they are closely associated with ER stress^3^.

In responding to ER stress, the unfolded protein response (UPR) signaling pathway is rapidly initiated in cells. The UPR is activated by three ER transmembrane proteins, protein kinase R-like ER kinase (PERK), inositol requiring 1 (IRE1), and activating transcription factor 6 (ATF6). Under physiological conditions, the peptides of three stress sensors in the ER lumen are bound to the chaperone glucose-regulated protein 78 (GRP78). When ER stress occurs, GRP78 disassociates with sensors and binds to misfolding proteins, alleviating the burden of the ER and leading to activation of the three sensors responsible for activating the UPR response. The UPR contributes to cell survival or death depending on the conditions of ER stress. In mild-ER stress, PERK is activated to phosphorylate eukaryotic initiation factor 2α (eIF2α), which blocks intracellular protein synthesis by inhibiting mRNA translation to ease the ER load. IRE1 cleaves XBP1 mRNA to form XBP1s, then XBP1s work with ATF4-producing chaperones to promote protein folding and induce ER-associated degradation (ERAD). ATF6 is devoted to upregulating GRP78 and glucose-regulated protein 94 (GRP94). The UPR signaling cascade promotes the ability of the ER to restore homeostasis when encountering tolerable ER stress. In cases of persistent and severe ER stress, the UPR cannot resist stress and recover ER homeostasis. C/EBP homology protein (CHOP) upregulation, which is mediated by PERK, is considered a symbol of ER stress-induced apoptosis. IRE1α induces proinflammatory and proapoptotic protein expression. When the UPR fails to attenuate stress in unresolvable ER stress, the apoptotic signaling pathway is activated. Therefore, ER stress functions as a double-edged sword in various diseases. However, the molecular mechanism of switching the function of ER stress has not been elucidated.

Recently, ER stress has been proposed to be a switch between cell survival and death. Several studies have revealed molecular mechanisms that possibly participate in the shifting functional response to ER stress. BCL-2 family proteins, including proapoptotic and antiapoptotic members, act as specialized stress sentinels, and they activate molecular switches to transition between a prosurvival response and induction of apoptosis in response to irreversible stress^4,5^. In addition, researchers found that the loss of Klotho, a single-pass transmembrane protein that is expressed in multiple tissues^6^, is causally linked to ER stress-induced apoptosis and that overexpressing Klotho causes cells to be resistant to chemically induced ER stress^7^. Klotho could act as a molecular switch modulating ER stress signaling crosstalk between cell survival and death in the wound healing process^8^. Knockdown of E2F1, which belongs to the E2F family of transcription factors, increases the amount of cell death in response to ER stress. E2F1 plays a key role in the cell survival/death decision under ER stress^9^.

In our previous research, we demonstrated that in acute liver failure, peroxisome proliferator activated receptor alpha (PPARα) functions in ER stress-induced hepatocyte apoptosis^10^ and that PPARα also plays an important role in the activation of autophagy^11^. Thus, we propose a hypothesis that PPARα plays a key role in regulating the transition of cell fate towards survival or death in response to ER stress. Our results showed that PPARα is a pivotal molecule that regulates the functional transition from protection to apoptosis in response to ER stress. The PPARα-autophagy pathway plays a protective role in promoting cell survival, and the PPARα-CHOP pathway participates in severe ER stress-induced apoptosis.

## Materials and methods

### Cell culture and treatment

AML12 cells were purchased from American Type Culture Collection (ATCC, Manassas, VA, USA). The cells cultured in Dulbecco’s modified Eagle’s medium (DMEM, Thermo Fisher, Inc., Rockford, IL, USA) with 10% fetal bovine serum (FBS, Thermo Fisher, Inc.) and 1% Pen–Strep (PS, Thermo Fisher, Inc.). Tunicamycin (TM, 10 µg/ml, Sigma, St. Louis, MO, USA) or thapsigargin (TG, 1 µg/ml, Sigma) were diluted in medium for the treatment of cells; they were used at different times or different doses to induce different severities of ER stress. The cells were incubated with TM (20 µg/ml) for 6 h to induce mild-ER stress and with TM (20 µg/ml) for 24 h to induce severe ER stress. A specific siRNA for PPARα (5 nM, Jima, Suzhou, China) was transfected into cells 24 h before the 6-h TM (20 µg/ml) treatment, and the PPARα activator Wy-14643 (50 μM) was used to treat cells 2 h before the 24-h TM (20 µg/ml) treatment. A Lipofectamine 2000 kit (Invitrogen, ThermoFisher Scientific, Inc.) was used to transfect specific siRNAs according to the manufacturer’s instruction. The autophagy inhibitor 3-methyladenine (3-MA, 5 mM, Sigma) was dosed into medium 1 h before TM administration, and the autophagy activator rapamycin (10 µg/ml, Sigma) was incubated with cells for 3 h. The specific siRNA for CHOP (5 nM, Jima) was transfected into cells to silence the expression of CHOP before TM administration. The lentivirus overexpression vector for CHOP or a negative control (N.C.) vector was transferred into cells at the concentration of 1 × 10^6^/ml for 48 h before TM administration. Polybrene reagent (5 μg/ml) was added to improve the transfection. The sequence of PPARα siRNA was 5′ GGAGCUGCAAGAUUCAGAATT 3′, and the sequence of COHP siRNA was 5′ CUCUCCAGAUUCCAGUCAGdTdT 3′. The efficacy of the siRNA and lentivirus treatments was confirmed by immunoblotting.

### Real-time reverse transcription-polymerase chain reaction (RT-PCR) assays

TRIzol reagent was used to extract total RNA from cells and liver tissue. Total RNA was reverse-transcribed to produce cDNA with a Superscript^TM^ III First-Strand Synthesis system (Invitrogen, Carlsbad, CA, USA). Reactions were then set up in 10 μl total volumes, and they contained 1× SuperMix (Platinum SYBR Green qPCR kit; Invitrogen), cDNA 2 μl and 0.5 μΜ of each primer. The reaction conditions were as follows: 50 °C for 2 min and 95 °C for 5 min, then 50 cycles of 95 °C for 15 s and 60 °C for 30 s. The relative level of target mRNA expression was normalized to HPRT and was analyzed using the 2^−ΔΔCt^ method.

### Immunoblotting assays

RIPA buffer contains protease and phosphatase inhibiters was used to extract protein from AML12 cells and mice liver tissue. extract protein from AML12 cells and mouse liver tissue. Protein quantification was performed using a bicinchoninic acid (BCA) protein assay kit (Biomed, Beijing, China) according to the manufacturer’s instructions. Protein was separated by sodium dodecyl sulfate-12% polyacrylamide gel electrophoresis and was subsequently transferred overnight to PVDF membranes at 4 °C. Monoclonal antibodies against PPARα (1:1000, Abcam, Cambridge, MA, USA), GRP78, CHOP, Bcl-2-related X-protein (Bax), B-cell lymphoma-extra-large (BCL-xL), caspase-3, cleaved-caspase-3 (1:1000, Cell Signaling Technology, Danvers, MA, USA) were diluted in TBST buffer with 5% skim milk, and then they were incubated at 4 °C overnight with the membranes. The next day, the membranes were washed in TBST buffer for 90 min, and then they were incubated with horseradish peroxidase-conjugated secondary antibody (1:2000, Cell Signaling Technology) for 60 min. An enhanced chemiluminescence system (Thermosphere, Inc.) was used to develop the band by exposure on an X-ray system.

### LDH and CCK-8 assays

AML12 cells were plated at a density of 1 × 10^4^ per well in a 96-well plate and were grown overnight. Cells were treated as previously described. Detection of the rate of cell death was performed with an LDH cytotoxicity assay kit (Beyotime, Shanghai, China) according to the manufacturer’s instructions. A cell counting kit-8 assay (Beyotime) was used to detect cell viability.

### Flow cytometry

An Annexin V-Phycoerythrin (PE)/7-Amino-Actinomycin (7-AAD) double staining assay (BD Bioscience, Franklin Lakes, NJ, USA) was used to detect apoptosis. AML12 cells were plated at a density of 1 × 10^6^ cells/well in 6-well plates. After exposure to drug treatment, cells were resuspended in 1× binding buffer and then were stained with PE and 7-AAD reagent for 15 min in the dark. The samples were analyzed by a FACScan flow cytometer (BD Bioscience), and the data were analyzed with FlowJo software.

### Animal treatments

For this study, 8- to 10-week-old male C57BL/6 mice were purchased from Capital Medical University (CMU, Beijing, China). The mice were kept in the CMU animal facility and had ad libitum access to food and water. All experiments were performed strictly in accordance with the ethical guidelines of the Capital Medical University Animal Experimentation Committee and were in full compliance with the National Institutes of Health Guide for the Care and Use of Laboratory Animals.

The animals were randomly divided into specific groups. The appropriate sample size was used to ensure the credibility of results. One group of mice were intraperitoneally injected with TM (Sigma, St. Louis, MO, USA) at a concentration of 1.0 mg/kg over a range of time (3, 6, 12, and 24 h), another was injected with the drug in various concentrations (1.25, 2.50, 5.00, and 10.00 mg/kg) for 24 h to create different phases and severities of ER stress. After drug treatment, liver tissues were harvested, frozen and stored in a freezer (−80 °C), and serum samples were collected for further research.

### Liver function

Liver function was assessed by analyzing the levels of alanine aminotransferase (ALT) and aspartate aminotransferase (AST). Blood collected from the mouse abdominal aorta was measured by a multiparametric analyzer (AU5400, Olympus, Japan) according to the automatic analysis program.

### Immunofluorescence assays

Frozen sections or cells on slides were fixed in paraformaldehyde for 10 min, washed in PBS buffer three times, and subsequently treated with Triton X-100 to permeabilize cell membranes. The sections were blocked in 10% goat serum and 3% BSA in PBS for 20 min. The following antibodies were incubated with the sections overnight at 4 °C: PPARα (1:500, Abcam), CHOP and LC3 (1:500, Cell Signaling Technology). After washing in PBS, the slides were incubated with Alexa Fluor 488 goat anti-rabbit IgG or Alexa Fluor 568 goat anti-mouse IgG (1:250, Invitrogen, ThermoFisher Scientific, Inc.) for 30 min at room temperature. The nuclei were stained with DAPI (1 µg/ml, Abcam) for 5 min. Finally, the images were analyzed by a Leica DM2500 fluorescence microscope.

### Statistical analysis

The results are presented as the mean with the standard deviation. Statistical analysis was conducted using unpaired t-tests or one-way analysis of variance (ANOVA), and a *P* value less than 0.05 is considered statistically significant.

## Results

### PPARα and autophagy-related genes expression profile at different phase of ER stress

**Fig. 1:**
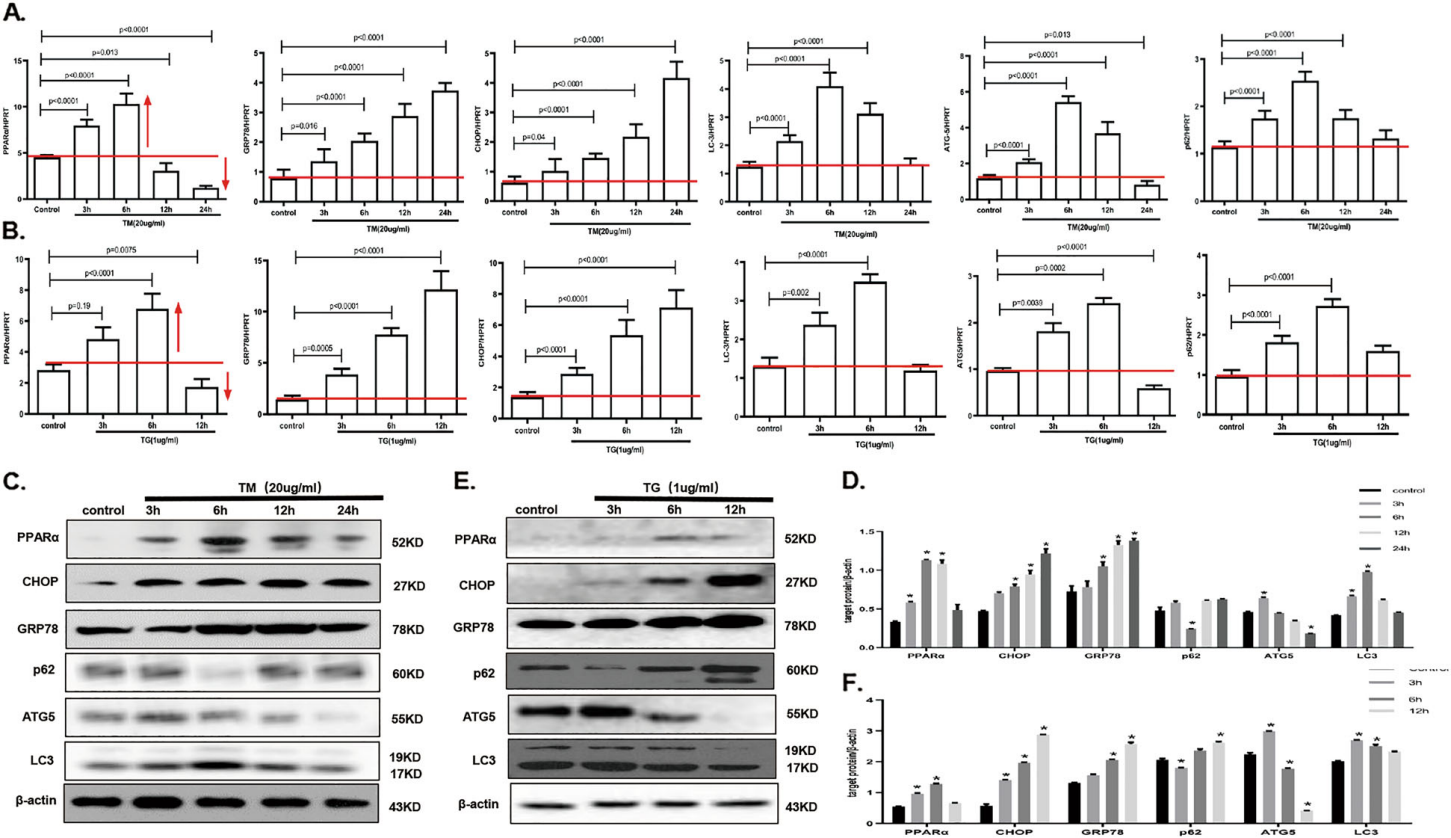
PPARα and autophagy-related genes expression profile at different phase of ER stress. Data are shown as mean SD of at least three independent experiments. **a** qRT-PCR assay for PPARα, GRP78, CHOP, and autophagy-related genes. AML12 cells were treated with PBS buffer or TM (20 µg/ml) for the indicated time points. **b** qRT-PCR assay for PPARα, GRP78, CHOP, and autophagy related genes. AML12 cells were treated with PBS buffer or TG (1 µg/ml) for the indicated time points. **c**, **d** Immunoblotting for PPARα, GRP78, CHOP, and autophagy related genes. AML12 cells was treated as in panel (**a**), the densitometry was measured by Image J. **e**, **f** Immunoblotting for PPARα, GRP78, CHOP, and autophagy-related genes. AML12 cells was treated as in panel (**b**), the densitometry was measured by Image J. *P < 0.0001.

AML12 cells were treated with TM at different times to induce different periods of ER stress. GRP78 and CHOP were used as markers to detect ER stress, and their levels gradually increased with ER stress, implying that the stress worsened. We observed that PPARα mRNA was significantly upregulated at the earlier stage of ER stress, at 6 h of treatment; however, its expression was decreased at the later stage of ER stress (Fig. 1a). Furthermore, we examined the mRNA levels of LC3, ATG5, and p62, which were activated at the early stage of ER stress but decreased at the late stage of ER stress (Fig. 1a). Furthermore, another ER stress inducer, TG, was used to induce ER stress at different times. Interestingly, the mRNA levels of PPARα, LC3, ATG5, and p62 showed similar expression profiles following TM administration (Fig. 1b). Next, we analyzed the protein levels of PPARα over different time periods of ER stress induced by TM. PPARα was also elevated at the early stage of ER stress and decreased at the late stage of ER stress. LC3 and ATG5 were also increased at the early stage and then decreased. With autophagy activation at the early stage of ER stress, the protein level of p62 was decreased, but p62 accumulated at the late stage of ER stress (Fig. 1c, d). The same results were also shown following the administration of TG (Fig. 1e, f). Next, we detected the protein expression of PPARα, CHOP and LC3. The results showed that PPARα was increased in the early stage of ER stress and decreased in the late stage of ER stress; CHOP expression increased with time, and LC3 was activated in the early stage of ER stress and decreased in the late stage of ER stress (Fig. S1). Therefore, PPARα and autophagy were activated under mild-ER stress conditions and inhibited under serious ER stress conditions.

### PPARα and autophagy-related gene expression profiles after exposure to different levels of ER stress

**Fig. 2:**
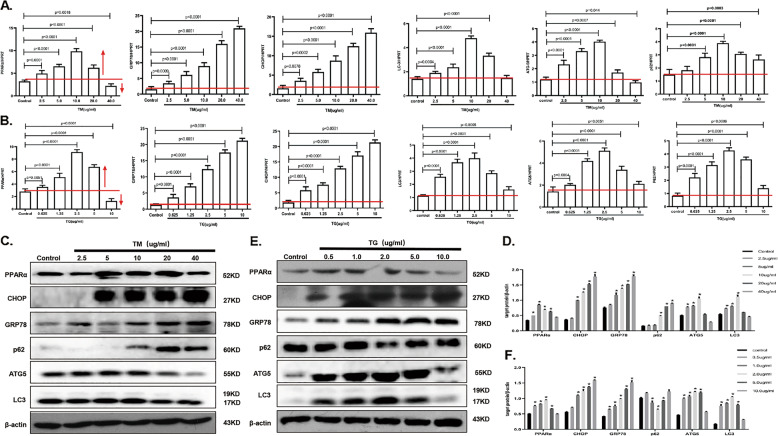
PPARα and autophagy-related gene expression profiles after exposure to different levels of ER stress. Data are shown as mean SD of at least three independent experiments. **a** qRT-PCR assay for PPARα, GRP78, CHOP, and autophagy related genes. AML12 cells were treated with PBS buffer or TM (24 h) for the indicated concentrations. **b** qRT-PCR assay for PPARα, GRP78, CHOP, and autophagy related genes. AML12 cells were treated with PBS buffer or TG (12 h) for the indicated concentrations. **c**, **d** Immunoblotting for PPARα, GRP78, CHOP, and autophagy related genes. AML12 cells was treated as in panel (**a**), the densitometry was measured by Image J. **e**, **f** Immunoblotting for PPARα, GRP78, CHOP, and autophagy related genes. AML12 cells was treated as in panel (**b**), the densitometry was measured by Image J. *P < 0.0001.

To further clarify the role of PPARα and autophagy-related genes after exposure to different levels of ER stress, we next investigated ER stress following treatment of cells with different concentrations of inducers. During low-dose TM induction, the mRNA levels of GRP78 and CHOP showed a slight increase compared with that of the control, suggesting that mild-ER stress was induced. During high-dose TM treatment, GRP78 and CHOP showed conspicuously high levels, suggesting that serious ER stress was induced. At the same time, PPARα mRNA was elevated significantly in the low-dose treatment and gradually suppressed as the concentration increased (Fig. 2a). In addition, the mRNA levels of LC3, ATG5, and p62 were activated during low-dose drug exposure and inhibited during high-dose drug exposure (Fig. 2a). In addition, TG was used as another ER stress inducer, and the target gene mRNA expression profile was the same as that induced by TM (Fig. 2b). Next, we detected the protein level of target molecules. We found that PPARα was increased under mild-ER stress and decreased under serious ER stress. In addition, ATG5 and LC3 protein levels were upregulated, while p62 decreased after low-dose TM exposure; however, ATG5 and LC3 decreased, while p62 accumulated after high-dose TM exposure (Fig. 2c, d). Following TG induction, we obtained the same results as those obtained after TM administration (Fig. 2e, f). Furthermore, we detected the expression of PPARα, CHOP, and LC3 with immunofluorescence, and the expression profile was consistent with previous findings (Fig. S2). Overall, during the transition from mild-ER stress to serious ER stress, we observed that PPARα and autophagy were promoted under mild-ER stress and suppressed under serious ER stress.

### Regulation of PPARα decides the fate of cells fate after exposure to different levels of ER stress

**Fig. 3:**
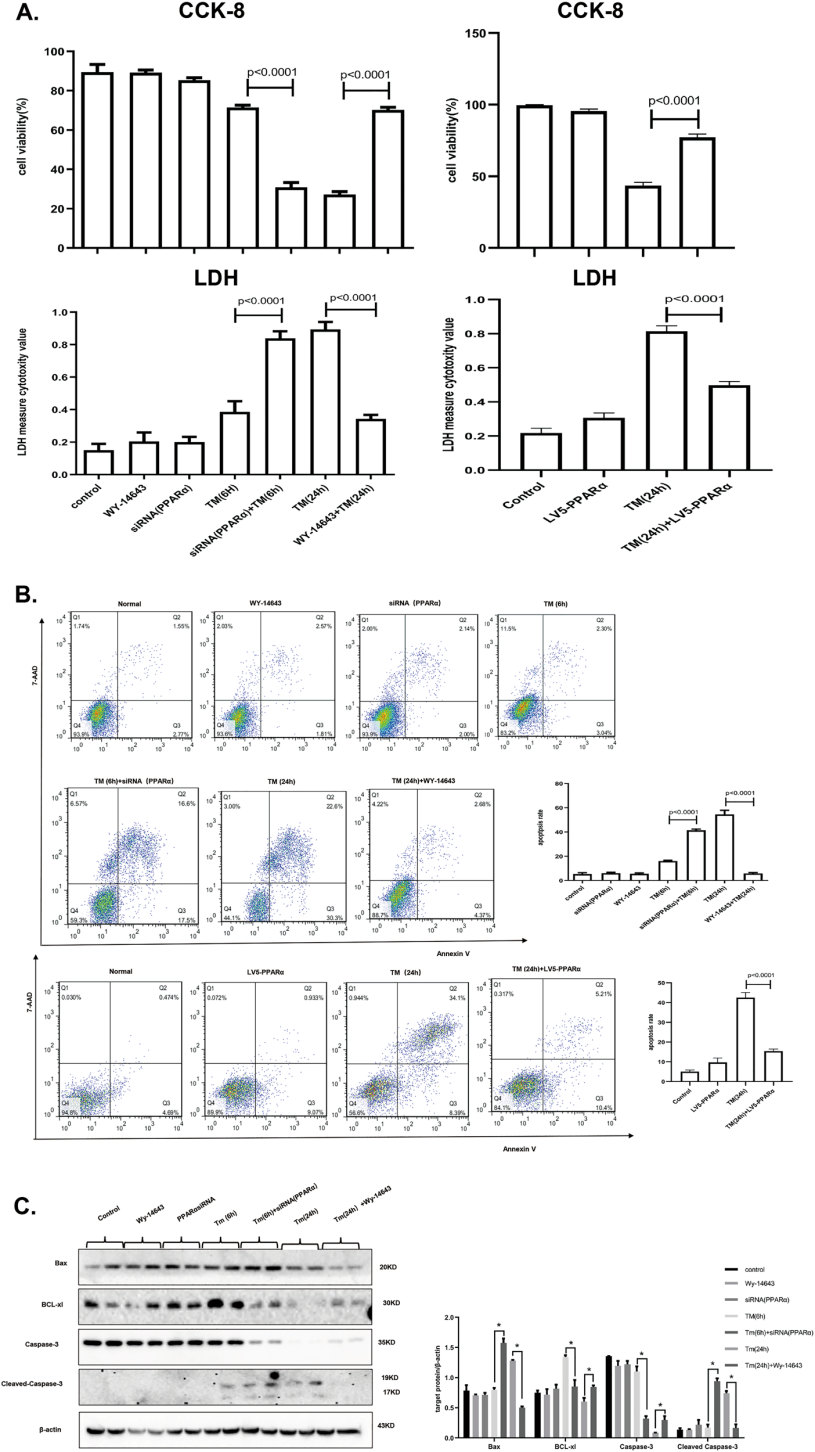
Regulation of PPARα decides the fate of cells fate after exposure to different levels of ER stress. Data are shown as mean SD of at least three independent experiments. **a** CCK-8 and LDH assay for cell viability and cell death measurement. AML12 cells were treated with DMSO or WY-14643 (50 µM) for 2 h, then stimulated by TM (20 µg/ml) for 24 h and transferred with siRNA of PPARα (5 nM) or control siRNA (5 nM) for 24 h, then treated with TM (20 µg/ml) for 6 h. AML12 cells were transferred with LV5-PPARα at first, then stimulated by TM (20 µg/ml) for 24 h. **b** Flow cytometry for cell apoptosis rate. AML12 cells was treated as in panel (**a**). The cells were recognized different populations in terms of their staining: the lower left quadrant was identified live cells, the lower right quadrant was identified early apoptosis cells, the upper left quadrant was identified necrotic cells, and the upper right quadrant was identified late apoptosis cells. The apoptosis rate was quantified by sum of early and late apoptosis. **c** Immunoblotting for Bax, BCL-XL, caspase-3, cleaved caspase-3. AML12 cells was treated as in panel (**a**). The densitometry was measured by Image J. *P < 0.0001.

Next, we explored the role of PPARα in response to different levels of ER stress. AML12 cells were treated with TM for 6 h to induce mild-ER stress and 24 h to induce serious ER stress. The siRNA was used to knockdown PPARα in mild-ER stress conditions, we used WY-14643 was to activated PPARα and lentivirus vector to upregulate PPARα, respectively in serious ER stress conditions. The effect of PPARα siRNA treatment was detected by immunoblotting (Fig. S5). Knockdown of PPARα promoted cell injury under mild-ER stress conditions, and activation of PPARα under serious ER stress conditions evidently increased cell survival. The cell death rate measured by LDH assay also proved the same results (Fig. 3a). Furthermore, we used flow cytometry and TUNEL assay, found that knocking down PPARα under mild-ER stress conditions increased ER stress-induced apoptosis, while activating PPARα under serious ER stress conditions significantly decreased ER stress-induced apoptosis (Figs. 3b and S6). Furthermore, we examined the protein levels of caspase-3, cleaved-caspase-3, Bax, and BCL-XL and found that cleaved-caspase-3 and Bax were significantly upregulated, while BCL-XL was reduced under mild-ER stress conditions following PPARα knockdown. Cleaved-caspase-3 and Bax were decreased, and BCL-XL was increased under serious ER stress conditions (Fig. 3c). Therefore, inhibition of PPARα significantly aggravated cell injury under mild-ER stress conditions, while activation of PPARα relieved serious ER stress-induced apoptosis.

### Mild-ER stress mediates the ability of PPARα to restore cell homeostasis via autophagy activation, and serious ER stress inhibits the ability of PPARα to promote apoptosis via autophagy impairment

**Fig. 4:**
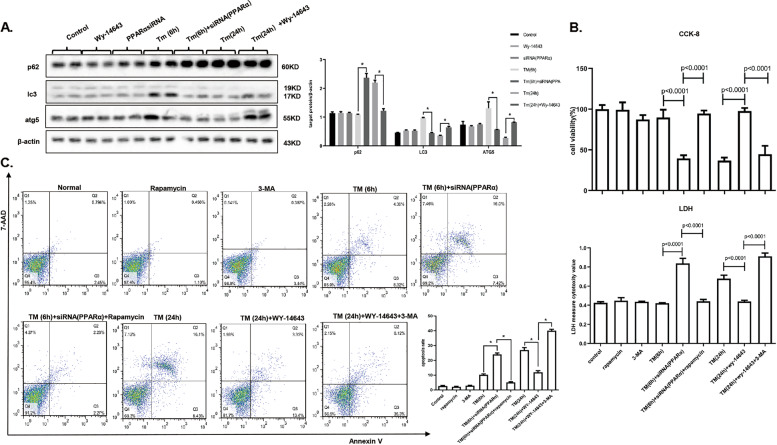
Mild-ER stress mediates the ability of PPARα to restore cell homeostasis via autophagy activation, and serious ER stress inhibits the ability of PPARα to promote apoptosis via autophagy impairment. Data are shown as mean SD of at least three independent experiments. **a** Immunoblotting for p62, lc3, and ATG5. AML12 cells were treated with DMSO or WY-14643 (50 µM) for 2 h, then stimulated by TM (20 µg/ml) for 24 h and transferred with siRNA of PPARα (5 nM) or control siRNA (5 nM) for 24 h, then treated with TM (20 µg/ml) for 6 h. **b** CCK-8 and LDH assay for cell viability and cell death measurement. AML12 cells were first transferred with siRNA of PPARα (5 nM) or control siRNA, then treated with TM (20 µg/ml) for 6 h and DMSO or rapamycin (10 µg/ml) for 3 h. Another group was stimulated with DMSO or WY-14643 (50 µM) for 2 h, 3-MA (5 mM) for 1 h, then treated by TM (20 µg/ml) for 24 h. **c** Flow cytometry for cell apoptosis rate. AML12 cells was treated as in panel (**b**). The analysis methods were as previously describe. The apoptosis rate was quantified by sum of early and late apoptosis. *P < 0.0001.

PPARα was knocked down under mild-ER stress conditions, and ATG5 and LC3 protein expression was low, while p62 accumulated; these results indicated that the autophagy process was impaired. In serious ER stress conditions, where PPARα was activated, ATG5 and LC3 protein expression was also activated, and p62 was decreased, indicating that autophagy was promoted (Fig. 4a). In conditions of mild-ER stress, which inhibited PPARα, rapamycin was used to activate autophagy, and the results showed that cell survival was significantly improved and that mortality and the cell apoptosis rate were decreased. In addition, under serious ER stress conditions, where PPARα was activated, autophagy was blocked by 3-MA, leading to a decrease in the cell survival rate and a significant increase in the death and apoptosis rates (Fig. 4b, c). The inhibition of autophagy counteracts the protection of PPARα activation, and autophagy activation abolishes PPARα inhibition injury. Thus, the protective effect of PPARα during mild-ER stress was mediated by the promotion of autophagy.

### Serious ER stress downregulates PPARα to promote apoptosis via CHOP activation, and mild-ER stress upregulates PPARα to restore homeostasis via CHOP inhibition

**Fig. 5:**
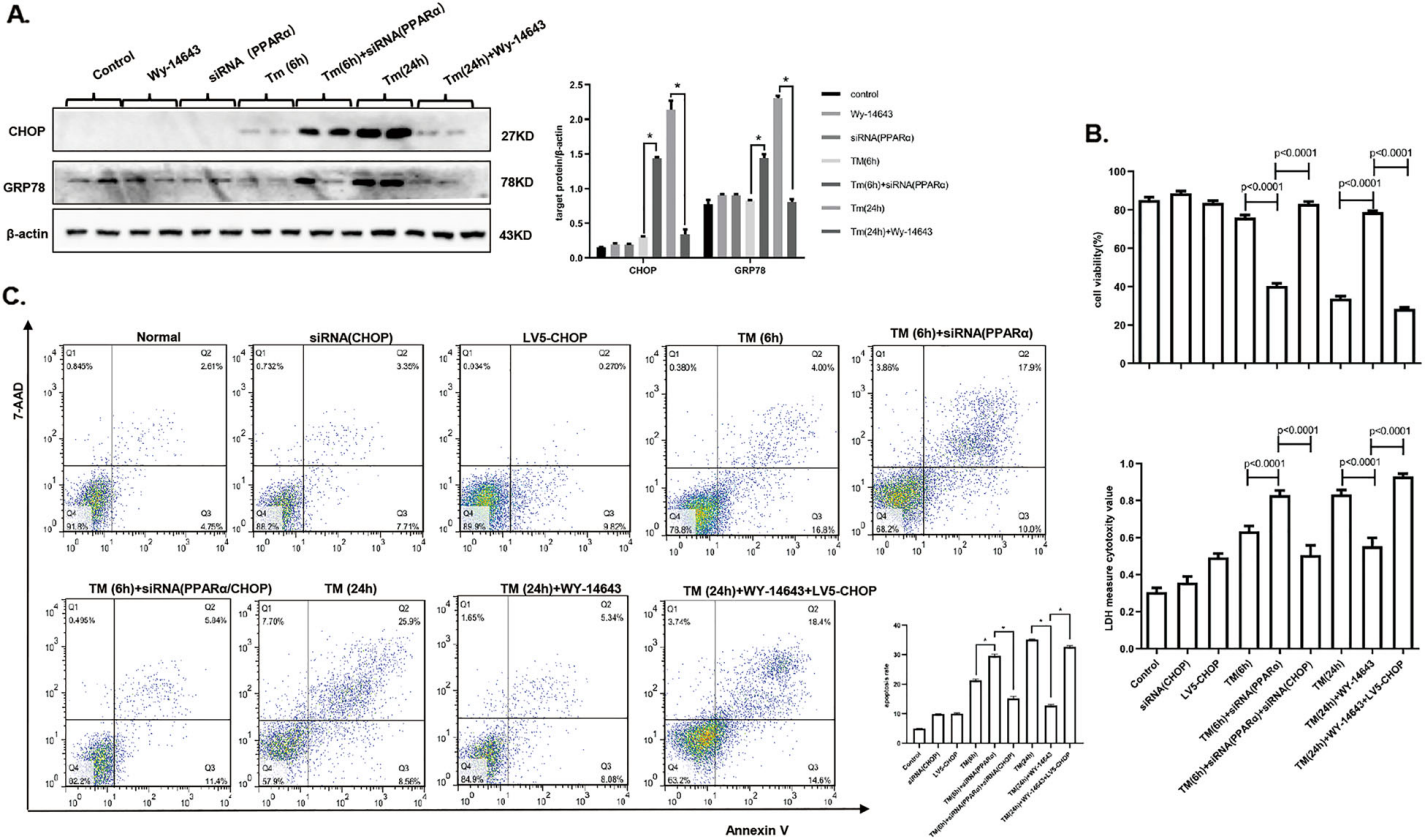
Serious ER stress downregulates PPARα to promote apoptosis via CHOP activation, and mild-ER stress upregulates PPARα to restore homeostasis via CHOP inhibition. Data are shown as mean SD of at least three independent experiments. **a** Immunoblotting for CHOP, GRP78. AML12 cells were treated with DMSO or WY-14643 (50 µM) for 2 h, then stimulated by TM (20 µg/ml) for 24 h and transferred with siRNA of PPARα (5 nM) or control siRNA (5 nM) for 24 h, then treated with TM (20 µg/ml) for 6 h. **b** CCK-8 and LDH assay for cell viability and cell death measurement. AML12 cells were first transferred with siRNA of PPARα (5 nM) and CHOP (5 nM) and control siRNA, then treated with TM (20 µg/ml) for 6 h and DMSO. Another group was transferred with LV5-chop and LV5-N.C. at first, then stimulated with DMSO or WY-14643 (50 µM) for 2 h, then treated by TM (20 µg/ml) for 24 h. **c** Flow cytometry for cell apoptosis rate. AML12 cells was treated as in panel (**b**). The apoptosis rate was quantified by sum of early and late apoptosis. *P < 0.0001.

We next examined the expression level of CHOP in cells with PPARα knocked down in mild-ER stress conditions and in serious ER stress conditions where PPARα was activated. The results show that CHOP was upregulated in the former group and downregulated in the latter group (Fig. 5a). Then, in the mild-ER stress conditions with knockdown of PPARα, CHOP was blocked by treatment with an siRNA, and there was an evident improvement in the cell viability rate and a decrease in the death rate and apoptosis rate. Under serious ER stress, in which PPARα was activated, CHOP was overexpressed, resulting in a decline in the cell survival rate and an increase in the death rate and apoptosis rate (Fig. 5b, c). The effect of CHOP siRNA treatment was shown, and TM treatment for 24 h was used as a positive control. Lentivirus-mediated CHOP overexpression was also detected, and PPARα expression was not affected by overexpression of CHOP (Fig. S5). This result shows that inhibition of CHOP could protect the cell from injury caused by mild-ER stress with inhibition of PPARα, and overexpression of CHOP could promote cell injury in serious ER stress, wherein PPARα is activated.

### PPARα and autophagy-related gene expression profiles at different times of TM-induced ER stress in a mouse model

**Fig. 6:**
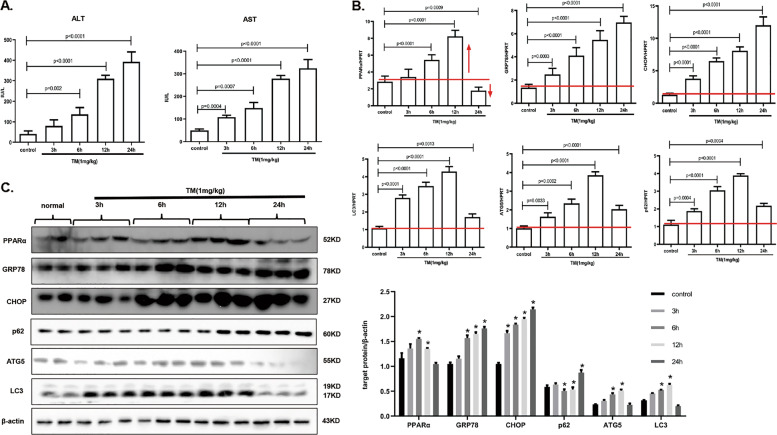
PPARα and autophagy-related gene expression profiles at different times of TM-induced ER stress in a mouse model. The experimenter was was blinded to the group allocation during the experiment. Data are shown as mean SD of at least three independent experiments. **a** AST, ALT measurement for liver function. Mice were injected with saline or TM (1 mg/kg) for the indicated time points. **b** qRT-PCR assay for PPARα, GRP78, CHOP, and autophagy-related genes. The mice were treated as in panel (**a**). **c** Immunoblotting for PPARα, GRP78, CHOP, and autophagy related genes. The mice were treated as in panel (**a**), the densitometry was measured by Image J. *P < 0.0001.

To verify this in vitro phenomenon, we next explored the role of PPARα in the functional conversion of ER stress in vivo. We first examined liver function by analyzing AST and ALT. With prolonged ER stress, the liver injury of animals worsened (Fig. 6a). Next, the mRNA of target genes was analyzed during times of ER stress. CHOP and GRP78 gradually increased, and PPARα was upregulated in the early stage and were downregulated in the late stage. These results were consistent with those of the in vitro experiment. In addition, autophagy was activated at the early stage and suppressed at the late stage (Fig. 6b). Furthermore, the protein level of PPARα showed the same trends that were observed in the mRNA data. ATG5 and LC3 were upregulated, and p62 was decreased at an early stage, suggesting that autophagy was activated. ATG5, LC3 were decreased, and p62 was accumulated at a late stage, suggesting that autophagy was impaired (Fig. 6c). Immunofluorescence detection of PPARα, CHOP and LC3 also exhibited a phenomenon that was similar to what was observed in vitro (Fig. S3). As mice were exposed to different severities of ER stress, we saw that PPARα and autophagy were activated under mild-ER stress and inhibited under serious ER stress.

### PPARα and autophagy-related gene expression profiles at different doses of TM-induced ER stress in a mouse model

**Fig. 7:**
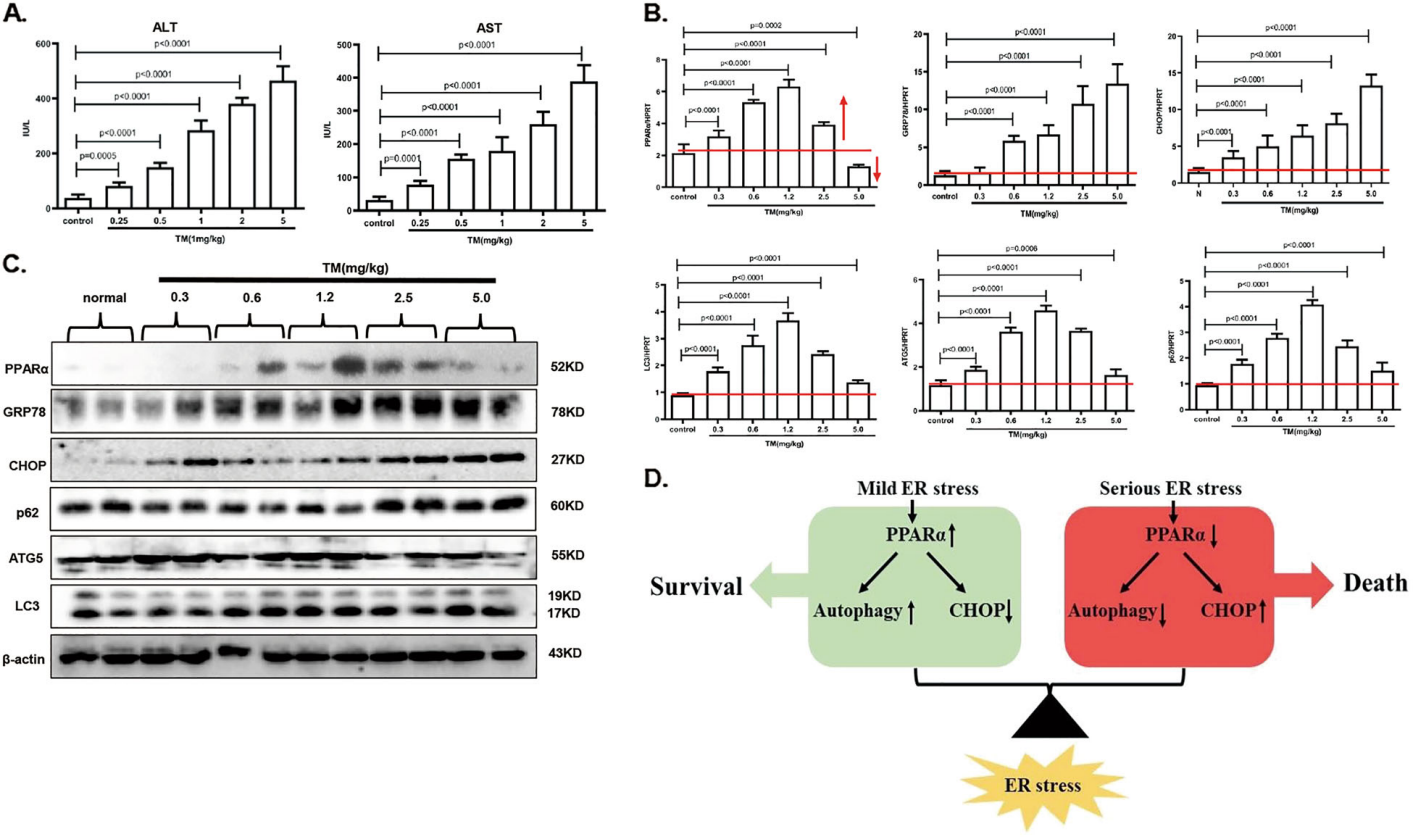
PPARα and autophagy-related gene expression profiles at different doses of TM-induced ER stress in a mouse model. The experimenter was was blinded to the group allocation during the experiment. Data are shown as mean SD of at least three independent experiments. **a** AST, ALT measurement for liver function. Mice were injected with saline or TM for 24 h the indicated concentrations. **b** qRT-PCR assay for PPARα, GRP78, CHOP, and autophagy-related genes. The mice were treated as in panel (**a**). **c** Immunoblotting for PPARα, GRP78, CHOP, and autophagy-related genes. The mice were treated as in panel (**a**), the densitometry was measured by Image J. *P < 0.0001. **d** In the different severities of ER stress, PPARα upregulation contributes to cell survival in the mild-ER stress via promoting autophagy and suppressing CHOP, PPARα downregulation contributes to cell apoptosis in the serious ER stress via inhibiting autophagy and promoting CHOP. PPARα is the balance fulcrum in the functional conversion of different severities of ER stress.

Then, different levels of ER stress were induced by treatment with different concentrations of TM. We first assessed the liver function of mice, which showed a gradual elevation of AST and ALT with increasing treatment concentrations (Fig. 7a). In addition, the mRNA levels of GRP78 and CHOP showed a slight increase under mild-ER stress conditions and a significant increase under serious ER stress conditions. However, the mRNA level of PPARα was elevated under mild-ER stress and reduced under serious ER stress. Furthermore, autophagy-related genes were activated under mild-ER stress conditions and decreased under serious ER stress conditions (Fig. 7b). In addition, protein levels were analyzed, and PPARα was at a high level under mild-ER stress conditions, but it was at lower levels under serious conditions. In addition, the autophagy factors ATG5 and LC3 were activated under mild-ER stress conditions and decreased under serious ER stress conditions. In addition, p62 degradation occurred under mild-ER stress conditions and accumulation occurred under serious ER stress conditions (Fig. 7c). In addition, with the increasing expression level of CHOP, PPARα shows an increase in mild-ER stress conditions and a decrease in serious ER stress conditions. LC3 was also activated under mild-ER stress conditions and suppressed under serious ER stress conditions (Fig. S4). Thus, PPARα and autophagy were activated under mild-ER stress conditions and inhibited under serious ER stress conditions.

## Discussion

Understanding the mechanisms of ER stress function conversion could enable the development of different strategies to control the function of ER stress at different stages of disease, thus providing a new strategy for disease prevention and treatment. In this study, with the transition of ER stress from mild to severe, PPARα was upregulated under mild-ER stress conditions but downregulated under serious ER stress conditions. Furthermore, we demonstrated that autophagy was activated under mild-ER stress conditions and inhibited under serious ER stress conditions. Severe ER stress directly led to impaired autophagy. ATG5 and LC3 expression decreased, and impaired autophagy attenuated p62 degradation, leading to its accumulation^12^. In mild-ER stress conditions, inhibition of PPARα resulted in a significant amount of apoptosis; anti-apoptosis genes were decreased, and pro-apoptosis genes were activated. Furthermore, the treatment led to autophagy blockade and CHOP overexpression. In serious ER stress conditions, activation of PPARα played a protective role in decreasing cell apoptosis. In addition, autophagy was activated, and CHOP was downregulated in this process. With the inhibition of PPARα in mild-ER stress conditions, knockdown of CHOP played a significant protective role in reducing apoptosis and promoting cell survival. With the activation of PPARα in serious ER stress conditions, CHOP decreased cell viability. In addition, in mild-ER stress conditions, which decreased PPARα, promoting autophagy ameliorated cell damage; in serious ER stress conditions, which activated PPARα, suppressing autophagy could exacerbate cell injury. Finally, PPARα expression profile in different stress conditions was demonstrated in vivo. This study reveals that PPARα is a switch in ER stress function conversion; PPARα activation promotes autophagy to relieve ER stress and promote cell survival, and PPARα inhibition upregulates CHOP to promote apoptosis.

PPARα, the first member of a subfamily of nuclear receptors (PPARs), also includes PPARβ and PPARγ. PPARs form a heterodimer with retinoid X receptor (RXR) to recognize specific DNA sequences and induce the expression of target genes^13^. PPARα is involved in a series of physiological processes, including mitochondrial fatty acid oxidation, catabolism, the inflammatory response, and the stress response. Recent studies have identified a possible role of PPARα in the ER stress response. Research has shown that activated PPARα exerts a protective effect by inhibiting myocardial ischemia/reperfusion injury-induced ER stress^14^. Further, PPARα can suppress the transcription of ATF2 to reduce ER stress-induced apoptosis in adipocytes^15^, and activation of PPARα can protect against increased ER stress to ameliorate hepatic insulin resistance as well as lipid accumulation^16^. Overall, multiple studies have shown that PPARα activation plays a protective role in regulating ER stress during disease development. Our data further demonstrated the subtle regulation of PPARα activation in mild-ER stress conditions promotes cell survival, and PPARα inhibition in serious ER stress conditions promotes apoptosis.

Autophagy is an adaptive cellular process that plays a “housekeeping” role in physiological conditions through lysosomal-dependent pathways that digest intracellular organelles and remove damaged cellular components^17^. In response to ER stress, autophagy is activated to remove ubiquitous unfolded/misfolded proteins and reduce ER stress^18,19^. However, there are studies showing that excessive autophagy can lead to aggravated cell injury^20^. The effects of autophagy induced by ER stress have a dual role, and they depend on the extent of ER stress^21^. Our data demonstrate that autophagy is activated in mild-ER stress conditions and impaired in serious ER stress conditions, providing a potential mechanism for the dual role of autophagy in ER stress. As a transcription factor, PPARα regulates numerous target genes that participate in cell adaptive procedures in response to stress. Studies have shown that the nuclear receptors PPARα regulate autophagy by controlling transcription of genes involved in autophagy pathways^22^; autophagy activation mediated by the upregulation of PPARα could relieve acute liver failure in mice^23^. In this study, our results reveal that PPARα activation in mild-ER stress conditions promotes restoration of homeostasis via promotion of autophagy.

When excessive ER stress was found to overwhelm the UPR compensation capacity, apoptosis was initiated. CHOP was upregulated and accumulated in the nucleus during apoptosis induced by ER stress^24^. Our previous research has shown that PPARα inhibition mediates CHOP to promote the inflammatory response in acute liver failure^11^. Additionally, in NAFLD development, the progressive suppression of PPARα was associated with the upregulation of CHOP^25^. Our results show that PPARα inhibition induced apoptosis in serious ER stress via CHOP activation.

In summary, we verified that PPARα is a key mediator in the functional conversion of ER stress response. ER stress is known to contribute to nearly all forms of liver diseases, so it is important to recognize the function of ER stress; further studies are still needed to explore the more precise mechanism of ER stress response. Distinct regulation of ER stress function based on the different stages of disease development new insight into clinical treatment.

## Supplementary information

Supplement Figure Legends

Supplement Figure 1

Supplement Figure 2

Supplement Figure 3

Supplement Figure 4

Supplement Figure 5

Supplement Figure 6

## References

[1] Song MJ, Malhi H (2019). The unfolded protein response and hepatic lipid metabolism in non alcoholic fatty liver disease. Pharmacol. Ther.

[2] Chusri P (2016). HCV induces transforming growth factor beta1 through activation of endoplasmic reticulum stress and the unfolded protein response. Sci. Rep..

[3] Wang X (2006). Mechanism of arylating quinone toxicity involving Michael adduct formation and induction of endoplasmic reticulum stress. Proc. Natl Acad. Sci. USA.

[4] Pihan P, Carreras-Sureda A, Hetz C (2017). BCL-2 family: integrating stress responses at the ER to control cell demise. Cell Death Differ..

[5] Rojas-Rivera D, Caballero B, Zamorano S, Lisbona F, Hetz C (2010). Alternative functions of the BCL-2 protein family at the endoplasmic reticulum. Adv. Exp. Med. Biol..

[6] Boksha IS, Prokhorova TA, Savushkina OK, Tereshkina EB (2017). Klotho protein: its role in aging and central nervous system pathology. Biochem. Biokhimiia.

[7] Banerjee S (2013). Klotho ameliorates chemically induced endoplasmic reticulum (ER) stress signaling. Cell. Physiol. Biochem..

[8] Mytych J, Solek P, Koziorowski M (2019). Klotho modulates ER-mediated signaling crosstalk between prosurvival autophagy and apoptotic cell death during LPS challenge. Apoptosis.

[9] Pagliarini V (2015). Downregulation of E2F1 during ER stress is required to induce apoptosis. J. Cell Sci..

[10] Zhang L (2016). Peroxisome proliferator-activated receptor alpha acts as a mediator of endoplasmic reticulum stress-induced hepatocyte apoptosis in acute liver failure. Dis. Models Mech..

[11] Zhang X (2017). Peroxisome proliferator-activated receptor alpha mediates C/EBP homologous protein to protect mice from acute liver failure. Inflamm. Res..

[12] Zhang H (2019). DEAD box protein 5 inhibits liver tumorigenesis by stimulating autophagy via interaction with p62/SQSTM1. Hepatology.

[13] Kersten S (2014). Integrated physiology and systems biology of PPARalpha. Mol. Metab..

[14] Yuan J (2018). PPARalpha activation alleviates damage to the cytoskeleton during acute myocardial ischemia/reperfusion in rats. Mol. Med. Rep..

[15] Liu Z (2016). Adiponectin reduces ER stress-induced apoptosis through PPARalpha transcriptional regulation of ATF2 in mouse adipose. Cell Death Dis..

[16] Chan SM (2013). Activation of PPARalpha ameliorates hepatic insulin resistance and steatosis in high fructose-fed mice despite increased endoplasmic reticulum stress. Diabetes.

[17] Ravanan P, Srikumar IF, Talwar P (2017). Autophagy: the spotlight for cellular stress responses. Life Sci..

[18] Feng J (2019). Autophagy activated via GRP78 to alleviate endoplasmic reticulum stress for cell survival in blue light-mediated damage of A2E-laden RPEs. BMC Ophthalmol..

[19] Cheng YC, Chang JM, Chen CA, Chen HC (2015). Autophagy modulates endoplasmic reticulum stress-induced cell death in podocytes: a protective role. Exp. Biol. Med..

[20] Ciechomska IA, Gabrusiewicz K, Szczepankiewicz AA, Kaminska B (2013). Endoplasmic reticulum stress triggers autophagy in malignant glioma cells undergoing cyclosporine a—induced cell death. Oncogene.

[21] Song S, Tan J, Miao Y, Li M, Zhang Q (2017). Crosstalk of autophagy and apoptosis: Involvement of the dual role of autophagy under ER stress. J. Cell. Physiol..

[22] Lee JM (2014). Nutrient-sensing nuclear receptors coordinate autophagy. Nature.

[23] Ren F (2016). Inhibition of glycogen synthase kinase 3beta promotes autophagy to protect mice from acute liver failure mediated by peroxisome proliferator-activated receptor alpha. Cell Death Dis..

[24] Lu TH (2014). Arsenic induces reactive oxygen species-caused neuronal cell apoptosis through JNK/ERK-mediated mitochondria-dependent and GRP 78/CHOP-regulated pathways. Toxicol. Lett..

[25] Flister KFT (2018). Long-term exposure to high-sucrose diet down-regulates hepatic endoplasmic reticulum-stress adaptive pathways and potentiates de novo lipogenesis in weaned male mice. J. Nutritional Biochem..

