# Supplementary material for: The critical role of PPARα in the binary switch between life and death induced by endoplasmic reticulum stress
Source: Cell Death Dis. 2020 Aug 11;11(8):691. doi: 10.1038/s41419-020-02811-4 (PMC7443130; doi:10.1038/s41419-020-02811-4)
Supplement: Supplementary file 1 — Supplement Figure Legends [file 41419_2020_2811_MOESM1_ESM.docx]

**Supplement Figure Legends**

**S1. PPARα and autophagy relative genes expression profile at different phase of ER stress.**

(A) Immunofluorescence for PPARα, CHOP. AML12 cells were treated with PBS buffer or TM (20ug/ml) for the indicated time points.

(B) Immunofluorescence for LC3. The cells were treated as in panel (A).

**S2. PPARα, autophagy related genes expression profile in different severity ER stress.**

(A) Immunofluorescence for PPARα, CHOP. AML12 cells were treated with PBS buffer or TM in the indicated concentrations for 24 hours.

(B) Immunofluorescence for LC3. The cells were treated as in panel (A).

**S3. PPARα and autophagy related genes expression profile at different times of TM induced ER stress mice model.**

(A) Immunofluorescence for PPARα, CHOP. Mice were injected with saline or TM (1mg/kg) for the indicated time points.

(B) Immunofluorescence for LC3. The mice were treated as in panel (A).

**S4. PPARα and autophagy related genes expression profile in the different doses of TM induced ER stress mice model**.

(A) Immunofluorescence for PPARα, CHOP. Mice were injected with saline or TM in the indicated concentrations.

(B) Immunofluorescence for LC3. The mice were treated as in panel (A).

**S5. The effect of siRNA and lentivirus vector was confirmed by immunoblotting.** Data are shown as mean SD of at least three independent experiments.

(A) Immunoblotting for PPARα. The AML12 cells were treated by siRNA of PPARα or N.C. (5nM) for 24 hours.

(B) Immunoblotting for CHOP. The AML12 cells were transferred with siRNA of CHOP or N.C. (5nM) for 24 hours. Then treated with TM (20ug/ml) or PBS for 24 hours.

(C) Immunoblotting for CHOP. The AML12 cells were treated by lentivirus vector of CHOP or N.C. for 48 hours. The densitometry was measured by Image J. *P<0.0001

**S6. Regulation of PPARα decides the fate of cells fate after exposure to different levels of ER stress.**

TUNEL assay was used to confirm cell apoptosis. AML12 cells were treated with DMSO or WY-14643(50 uM) for 2 hours, then stimulated by TM (20ug/ml) for 24 hours and transferred with siRNA of PPARα (5nM) or control siRNA (5nM) for 24 hours, then treated with TM (20ug/ml) for 6 hours.
