# Supplementary figures and images for: The critical role of PPARα in the binary switch between life and death induced by endoplasmic reticulum stress
Source: Cell Death Dis. 2020 Aug 11;11(8):691. doi: 10.1038/s41419-020-02811-4 (PMC7443130; doi:10.1038/s41419-020-02811-4)

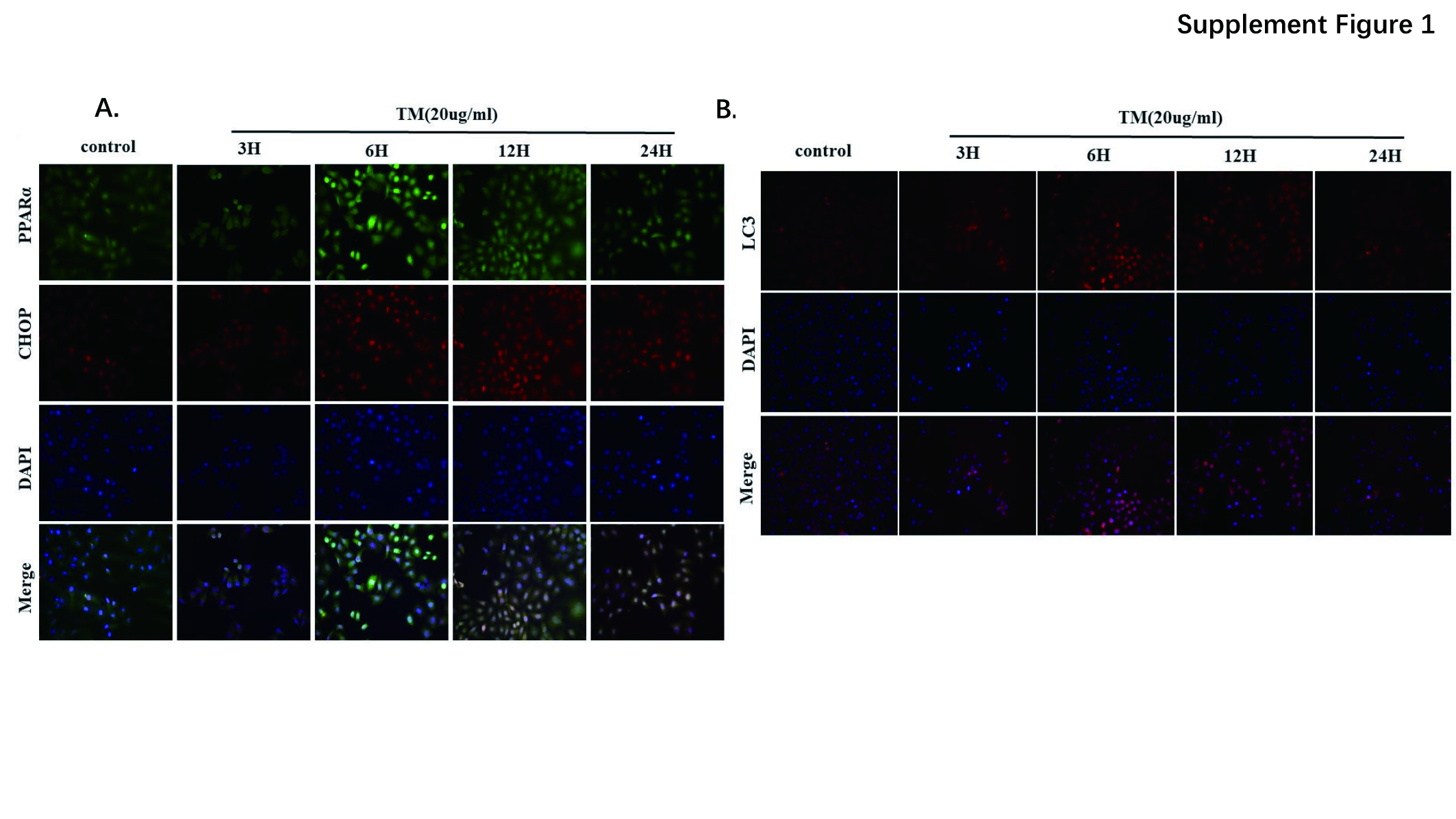

Supplement: Supplementary file 2 — Supplement Figure 1 [file 41419_2020_2811_MOESM2_ESM.tif]

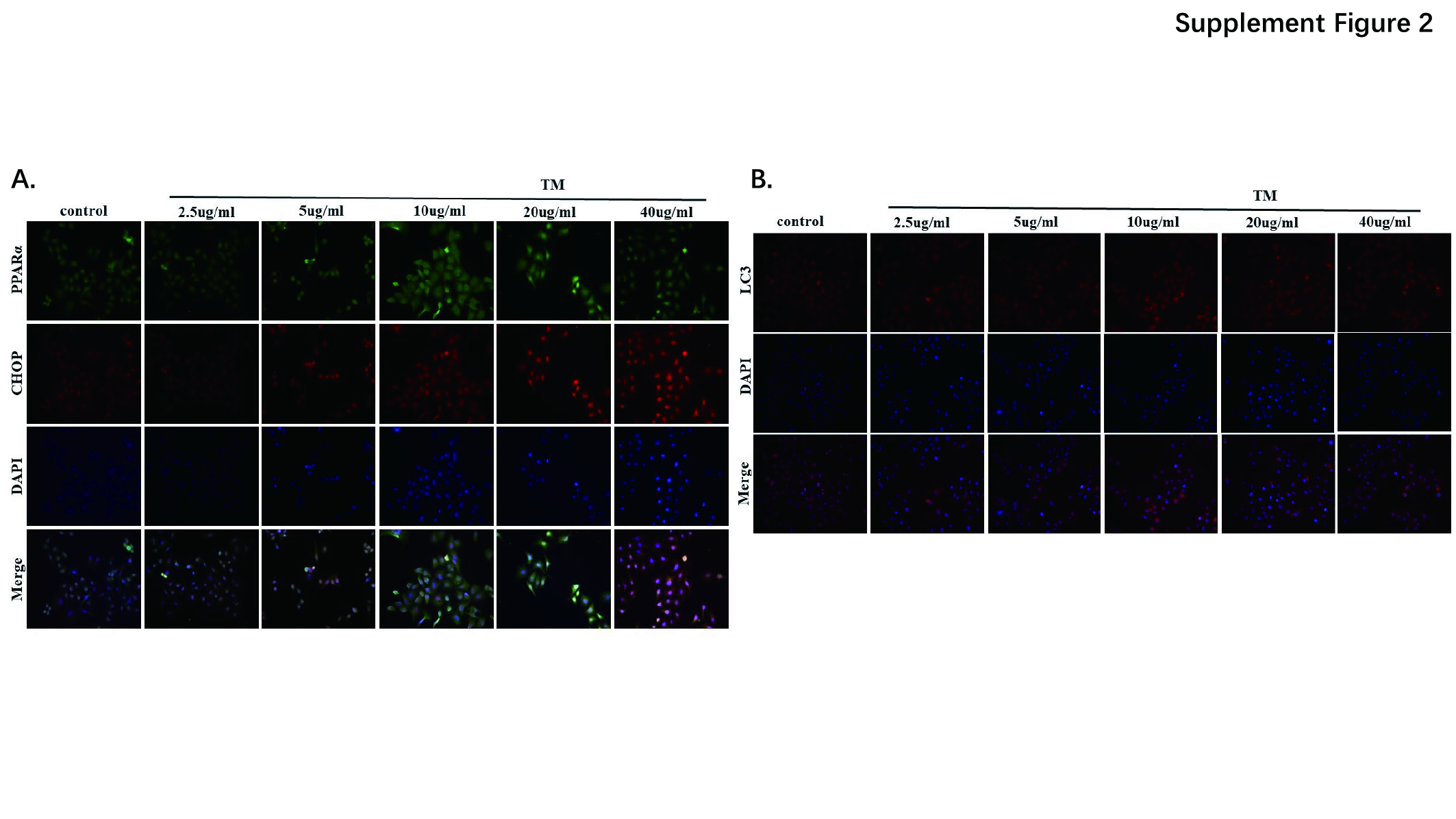

Supplement: Supplementary file 3 — Supplement Figure 2 [file 41419_2020_2811_MOESM3_ESM.tif]

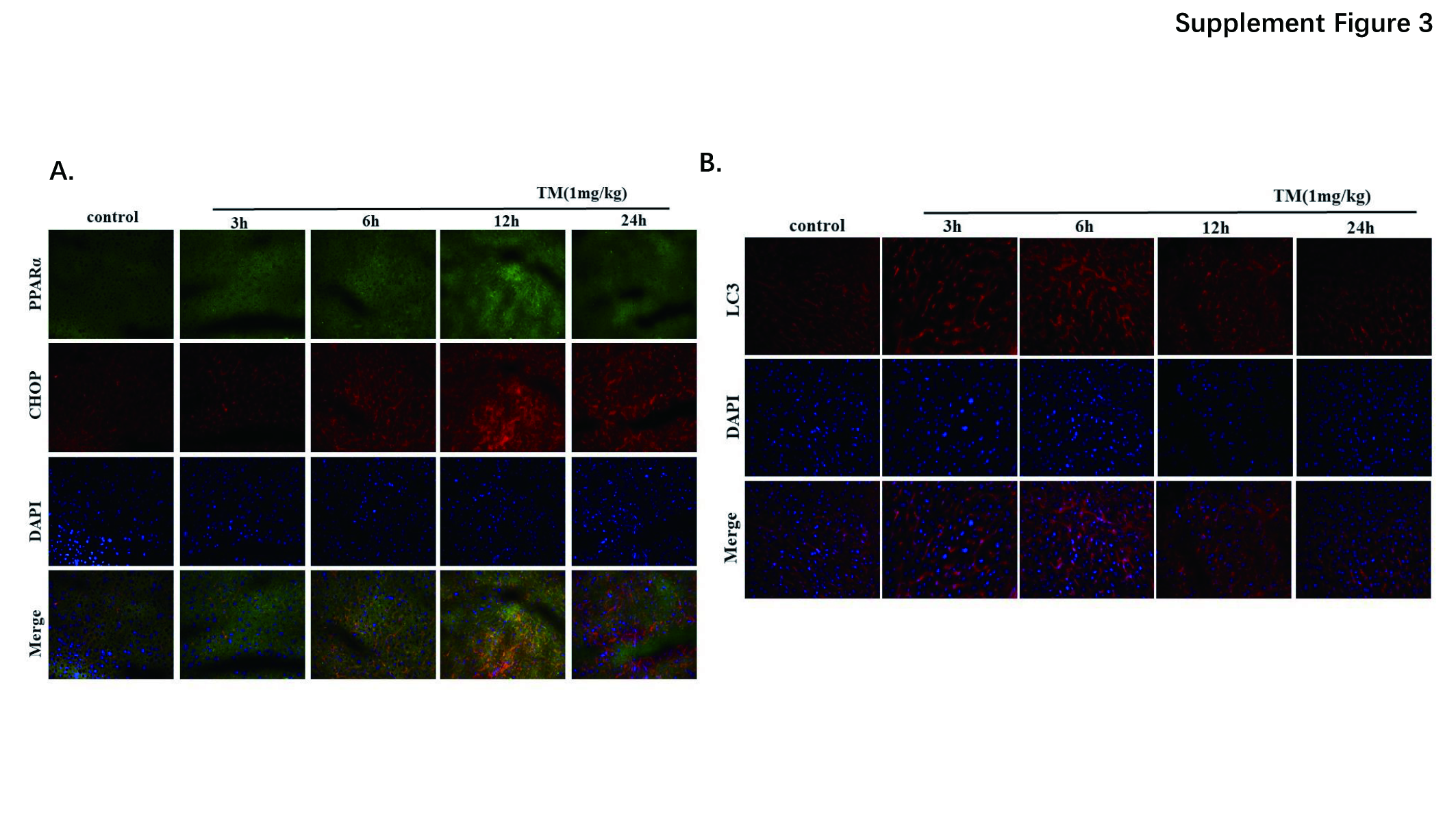

Supplement: Supplementary file 4 — Supplement Figure 3 [file 41419_2020_2811_MOESM4_ESM.tif]

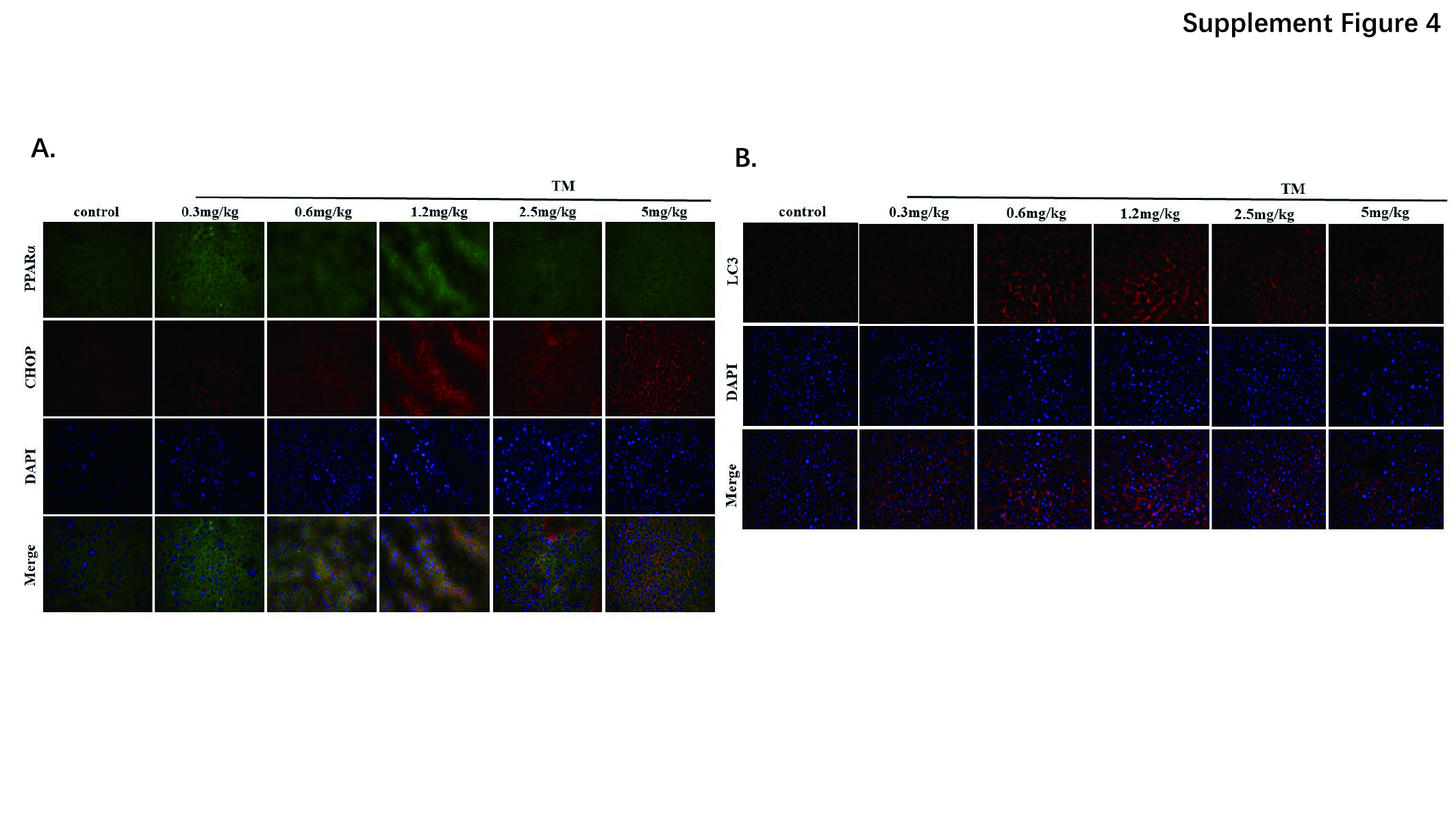

Supplement: Supplementary file 5 — Supplement Figure 4 [file 41419_2020_2811_MOESM5_ESM.tif]

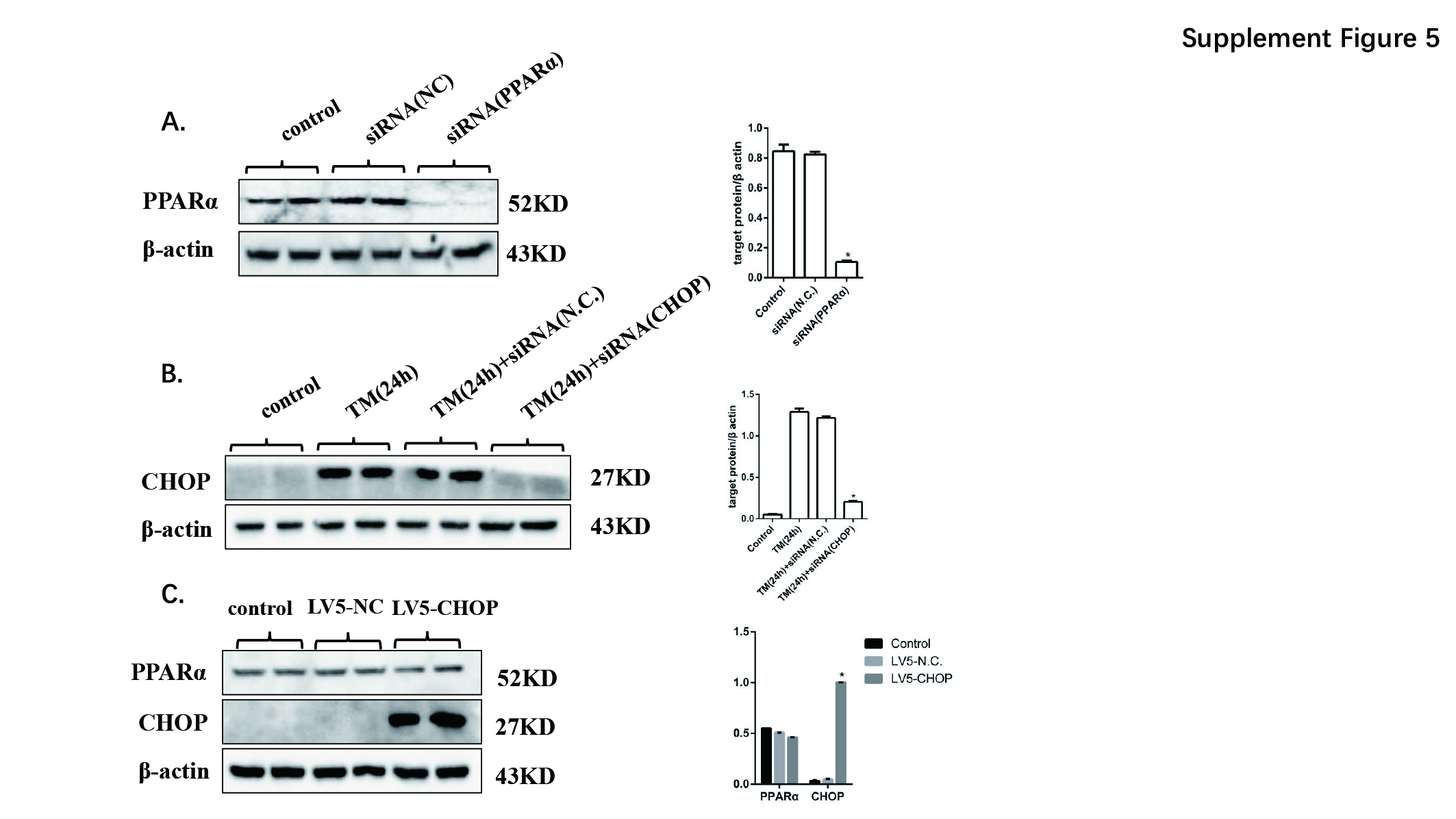

Supplement: Supplementary file 6 — Supplement Figure 5 [file 41419_2020_2811_MOESM6_ESM.tif]

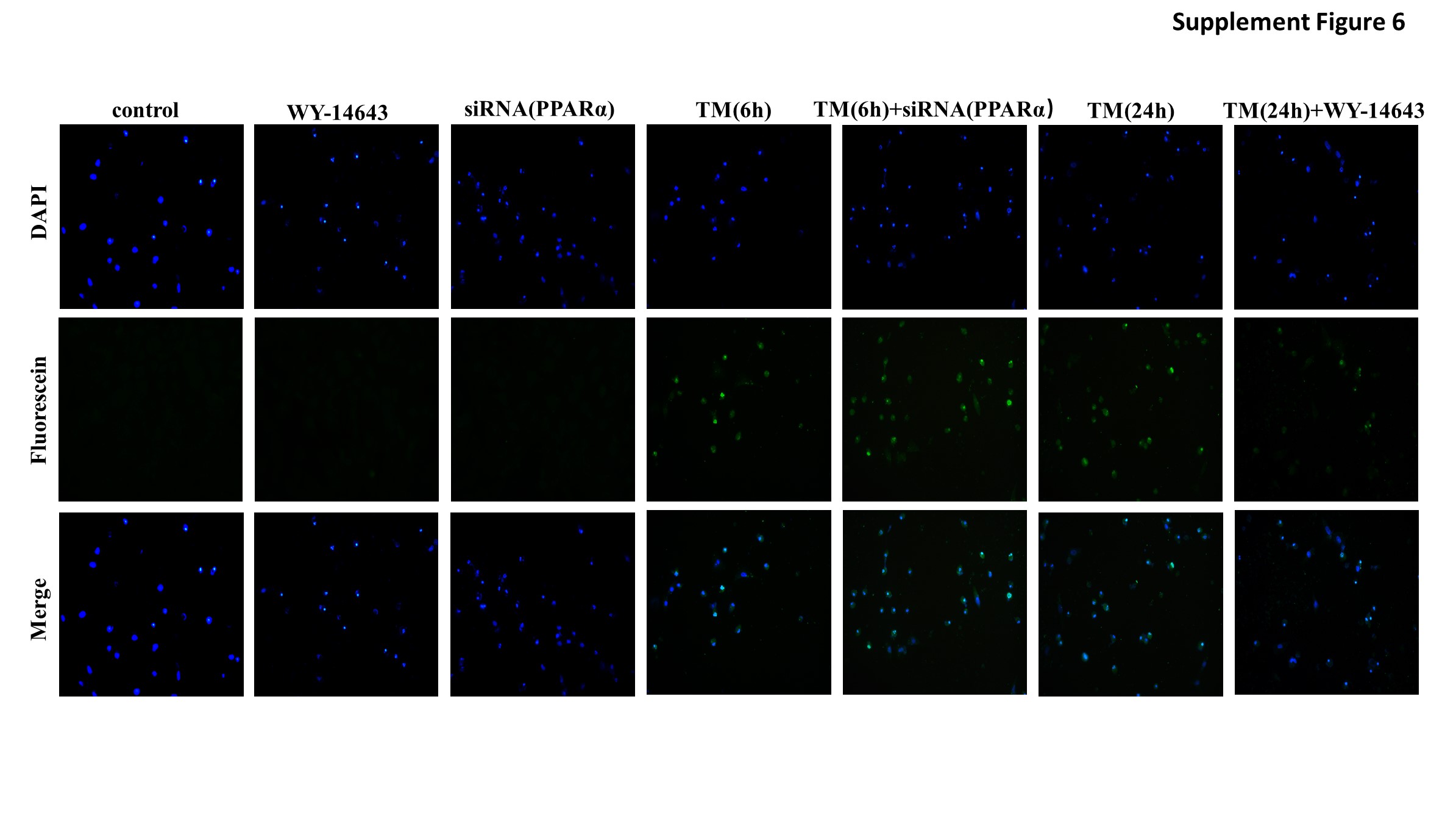

Supplement: Supplementary file 7 — Supplement Figure 6 [file 41419_2020_2811_MOESM7_ESM.jpg]
